# Plasma tRF-16-79MP9PD and tRF-28-OB1690PQR304 as potential biomarkers for 4- to 7-year-old children with obstructive sleep apnea-hypopnea syndrome

**DOI:** 10.3389/fped.2023.1141348

**Published:** 2023-05-30

**Authors:** Junhua Wu, Xiaohong Cai, Yanbo Lu, Yijing Shen, Zhisen Shen, Qin Lyv

**Affiliations:** ^1^ School of Medicine, Ningbo University, Ningbo, China; ^2^ Department of Pediatric, Ningbo Women and Children's Hospital, Ningbo, China; ^3^ Department of Otorhinolaryngology, Lihuili Hospital of Ningbo University, Ningbo, China

**Keywords:** obstructive sleep apnea-hypopnea syndrome, tRNA-derived fragments, biomarker, pediatrics, diagnostic screening

## Abstract

**Background:**

We investigated the expression and the potential value of plasma transfer RNA-derived fragments (tRFs) of children with obstructive sleep apnea–hypopnea syndrome (OSAHS) as screening biomarkers.

**Methods:**

At first, we randomly selected five plasma samples from the case group and the control group for high-throughput RNA sequencing. Secondly, we screened two tRFs with different expression between the two groups, amplified it by quantitative reverse transcription-PCR (qRT-PCR) on all samples. Then we analyzed the diagnostic value of the tRFs and their correlation with the clinical data.

**Results:**

A total of 50 OSAHS children and 38 healthy controls were included. Our results demonstrated that the plasma levels of tRF-16-79MP9PD and tRF-28-OB1690PQR304 were significantly down-regulated in OSAHS children. Receiver operating characteristic curve (ROC) showed that the area under the curve (AUC) of tRF-16-79MP9PD and tRF-28-OB1690PQR304 was 0.7945 and 0.8276. In addition, the AUC of the combination reached 0.8303 with 73.46% and 76.42% sensitivity and specificity. Correlation analysis showed that the degree of tonsil enlargement, hemoglobin (Hb) and triglyceride (TG). were related to the expression levels of tRF-16-79MP9PD and tRF-28-OB1690PQR304. Multivariable linear regression analysis showed that degree of tonsil enlargement, Hb and TG related to tRF-16-79MP9PD while degree of tonsil enlargement and Hb related to tRF-28-OB1690PQR304.

**Conclusions:**

The expression levels of tRF-16-79MP9PD and tRF-28-OB1690PQR304 in the plasma of OSAHS children decreased significantly which were closely related to the degree of tonsil enlargement, Hb and TG, may become novel biomarkers for the diagnosis of pediatric OSAHS.

## 1. Background

Obstructive sleep apnea-hypopnea syndrome (OSAHS) is a common disorder characterized by the snore and repeated episodes of partial or complete collapse of the upper respiratory tract during sleep (1). It has been widely accepted that hypoxia stress caused by upper airway obstruction in OSAHS can lead to systemic multiple system and organ damage (2). The main cause of upper airway obstruction in children is adenoid and/or tonsil hypertrophy (3). OSAHS can occur at all ages, with the peak of incidence occurring between 2 and 8 years old, and the most obvious clinical symptoms and surgical intervention mainly occur between 4 and 7 years old because of tonsil and adenoid hypertrophy accounting for the largest proportion of upper airway volume at this age (4, 5). The incidence of pediatric OSAHS is up to 1%–3% and is on the rise (6). OSAHS may lead to a series of pathological changes such as abnormal behavior, cognitive dysfunction, growth retardation and so on (7–10). In addition, many studies have shown that long-term OSAHS condition can increase the risk of diabetes, hypertension, metabolic diseases and cardiovascular diseases (11–13).

Transfer RNAs (tRNAs) are a class of non-coding RNAs that transport amino acids and assist protein synthesis (14). Under special conditions such as hypoxia, hunger and stress, pre- and mature tRNAs may generate new specific small RNA fragments after enzymatic splicing and chemical modification, which is called tRNA-derived small RNAs (tsRNAs) (15, 16). tsRNAs can also be mainly divided into tRNA halves and tRNA-derived fragments (tRFs) according to the cleavage loci and length (15). It has been reported that tRFs has a variety of biological functions, such as regulating translation level, affecting gene expression and inhibiting cell apoptosis (17). Recently, with the development of high-throughput sequencing and chip technology, novel tRFs have been gradually discovered and attracted researchers' interests. The relationship between tRFs and the occurrence of human diseases such as cancer and metabolic diseases has also been gradually revealed (18, 19).

Intermittent hypoxia is one of the typical characteristics of OSAHS, which can increase the expression of reactive oxygen species, resulting in systemic inflammation state, endothelial dysfunction and metabolic dysregulation (20). Therefore, biomarkers of hypoxia and oxidative stress may also be used to evaluate OSAHS. tsRNAs will be produced when cells are cleaved at specific tRNA sites in response to environmental stresses such as hypoxia, oxidative stress or viral infection (21). In recent years, several OSAHS-related differential microRNAs have been identified to be involved in a variety of pathophysiological processes (22, 23). Taken together, we speculate that tsRNAs may be related to the occurrence and development of OSAHS. In this study, we aim to explore the expression and the potential diagnostic screening value of plasma tRFs in children with OSAHS and further determine the association between tRFs and clinically relevant data in OSAHS children.

## 2. Methods

### 2.1. Research subjects

This study was an observational clinical study. We collected data from 50 patients aged 4–7 years old who were diagnosed with OSAHS after PSG based on medical history in Ningbo Women and Children's Hospital (Zhejiang, China) from November 2020 to December 2021. The diagnostic criteria of OSAHS refer to Chinese Guideline for the Diagnosis and Treatment of Childhood Obstructive Sleep Apnea (2020) (3), which diagnoses children with obstructive sleep apnea hypopnea index (OAHI) ≥1 events/h as OSAHS. Exclusion criteria for this study were children with congenital abnormalities of the nasolaryngology or airway, craniofacial malformation, anemia, digestive tract malformation, and those who had used antibiotics or probiotics in the last 3 months. In addition, 38 healthy control children were recruited as the control group and underwent free PSG examination.

In this study, basic information such as age, gender, body weight and height of all subjects were collected. Body mass index (BMI) was calculated by dividing weight by height squared.

This study has been approved by the ethical committee of Ningbo Women and Children's Hospital on November 5th, 2020 (Approval No. EC2020-047) and all participants have signed the informed written consent forms.

### 2.2. Specimen collection

All blood samples were collected from the peripheral venous blood of the children in Ningbo Women and Children's Hospital. For tsRNA studies, blood was firstly harvested into an ethylenediaminetetraacetic acid anticoagulation tube (EDTA). Within 1 h of blood collection, all specimens were centrifuged at 3,000 rpm/10 mins under 4°C to obtain plasma. The supernatant was transferred to RNase-free Eppendorf tubes and then stored at −80°C until RNA extraction.

### 2.3. tsRNA isolation and quality assessment

Total RNA, including tsRNA, was extracted and isolated from plasma samples by using TRIzol LS reagent (Invitrogen, Carlsbad, CA, USA) according to the instructions of manufacturer. RNA concentration was measured on NanoDrop™ Nd-1000 spectrophotometer (NanoDrop, Thermo Fisher Scientific, Inc., Wilmington, DE, USA) at 260 nm, 280 nm. The acceptance ratio standard for the study is 1.8–2.0. The extracted total RNA was stored at −80°C for subsequent experiments.

### 2.4. tsRNA pretreatment, small RNA library preparation and sequencing

In this study, plasma samples of 5 pairs of children with OSAHS and healthy controls were firstly selected. After extracting total RNA, small isolated RNA was processed with the rtStarTM tRF and tiRNA Pretreatment Kit (Arraystar, Rockville, MD, USA) to remove the superfluous modification groups. Furthermore, compared to the original RNA, pretreated RNA had been purified twice to make it meet the concentration requirements for subsequent experiments. Purified RNA was then sent to Shanghai Kangcheng Company (Shanghai, China) for constructing small RNA libraries and performing small RNA sequencing analysis. Briefly, according to the requirements of the commercial kit NEB Next® Multiplex Small RNA Library Prep Set for Illumina (New England BioLabs, Inc., Ipswich, MA, USA), the pretreated RNA was bound with 3′ and 5′ adapters, and small RNA library was established after adding reverse transcription primers to synthesize cDNA. The Illumina NextSeq 500 (Illumina, Inc., San Diego, CA, USA) system was then used for sequencing.

In the original sequencing data, low-quality reads and short reads (<15 nt) should be firstly filtered. After this step, all selected RNA reads were aligned to several non-coding RNA database, such as miRBase database (http://www.mirbase.org/), Genomic tRNA database (http://gtrnadb.ucsc.edu/), tRFdb (http://genome.bioch.virginia.edu/trfdb/) and MintBase (https://cm.jefferson.edu/MINTbase/). In the expression of tsRNAs between OSAHS and healthy controls, the clean data was measured after the expression level was normalized. We believed that tsRNAs with a fold change of ≥2.0 and *P* < 0.05 are significantly different expressions. We chose tRF-16-79MP9PD and tRF-28-OB1690PQR304 for follow-up research.

### 2.5. Quantitative reverse transcription-polymerase chain reaction (qRT-PCR) verification of tsRNA sequencing results

**Figure 1:**
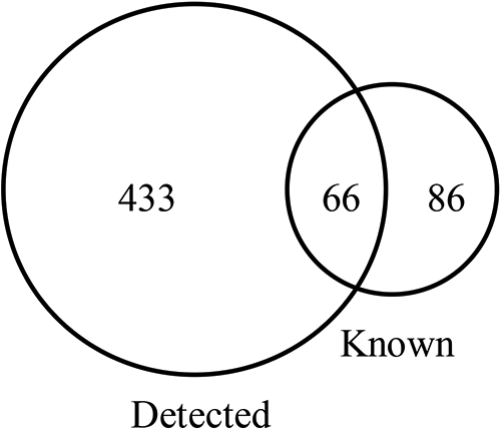
The number of tsRNAs detected in 5 OSAHS children and 5 healthy children plasma samples and the number of tsRNAs known in the tRFs database.

Two thousand nanograms of RNA were reverse-transcribed to cDNA according to the instruction of the rtStar™ First-strand cDNA Synthesis Kit (Arraystar, Rockville, MD, USA). First step, input 1 µl of 3′ Adaptor, 0.5 µl of RNA Spike-in and nuclease-free water into 2000 ng RNA to make a total volume of 8.8 µl, then incubate the mixture at 70°C for 2 min. Second, we added 7.2 µl of 3′ Ligation Reaction Buffer, 1 µl 3′ Ligation Enzyme Mix and 1 µl of RNase Inhibitor in sequence, which was then incubated for 1 h at 25°C. Then the procedure involves addition of a 1 µl of nuclease-free water and 1 µl of RT-primer, and incubation for 5 min at 75°C, 15 min at 37°C, 15 min at 25°C. Third, 1 µl of 5′Adaptor (denatured), 2.5 µl of 10 mM ATP, 0.5 µl of 5′ Ligation Reaction Buffer, 1 µl of 5′ Ligation Enzyme Mix and 20 µl samples were mixed and incubated at 25°C at 1 h. Lastly, 25 µl of Adaptor-Ligated RNA, 8 µl of First-Strand synthesis reaction buffer, 3 µl of 0.1 MDTT, 2 µl of 2.5 mM dNTP Mix, 1 µl of RNase Inhibitor and 1 µl of Reverse Transcriptase were mixed in a nuclease-free tube, and the sample was heated for 60 min at 45°C, and then to 75°C for 15 min. qRT-PCR was firstly conducted on plasma samples of the previous 5 pairs of children with OSAHS and healthy controls, and the sequencing results of PCR products showed complete matching with tsRNA original sequence (Figure 1). After that, qRT-PCR was used to detect more differences in the expression levels of OSAHS children and healthy children, so as to verify whether it was consistent with the previous sequencing results. The qRT-PCR reaction was performed on a Mx3005P Real-Time PCR System (Stratagene, Palo Alto, CA, USA). The PCR primer synthesis (Table 1) and sequencing of PCR products were done by Sangon Biotech (Shanghai, China). U6 was used as an internal reference gene, and the relative gene expression was calculated as ΔCT = CT^tRF ^− CT^U6^, Larger ΔCT values indicate lower expression.

**Table 1:** Primer sequences involved.

Name	Sequence of primer
U6	F:5′ GCTTCGGCAGCACATATACTAAAAT 3′
	R:5′ CGCTTCACGAATTTGCGTGTCAT 3′
tRF-16-79MP9PD	F:5′ TACAGTCCGACGATCGTTTCC 3′
	R:5′GTGCTCTTCCGATCTGCTACACTA 3′
tRF-28-OB1690PQR304	F:5′ ACAGTCCGACGATCGAAAAAGT 3′
	R:5′ ATCTCCAACCCCATGGCCT 3′

### 2.6. Statistical analysis

Statistical analysis was performed using SPSS version 24.0 and GraphPad Prism 8.0. The measurement data were expressed as mean ± standard deviation (*X* ± SD) and the counting data were expressed in percentage (%). independent samples *t*-test was used to evaluate the significance of measurement data between the two groups. *χ*^2^ test was used for comparison of counting data. Receiver operating characteristic (ROC) curve and area under the curve (AUC) were carried out to evaluate the diagnostic value of tRFs in OSAHS. Correlations between tRFs and relevant clinical parameters were analyzed by Pearson and Spearman correlation analysis. Multivariate linear regression analysis was performed to assess the association between plasma tRFs levels and several clinical parameters. All experiments were repeated at least three times independently. *P* value < 0.05 was considered statistically significant.

## 3. Results

### 3.1. Participants' characteristics

A total of 50 OSAHS children and 38 healthy controls were enrolled. Parameters of all subjects are summarized in Table 2. There were significant differences in OAHI and lowest oxygen saturation (LaSO_2_) between the OSAHS children and the controls. Besides, there were differences in height, serum creatinine (Scr) and total cholesterol (TC) between the two groups.

**Table 2:** All parameters of participants.

Groups	HC (*n* = 38)	OSAHS (*n* = 50)	*t/χ* ^2^	*P-*value
Age (years)	5.63 ± 0.75	5.35 ± 0.81	−1.667	0.0990
Male, *n* (%)	22 (58%)	27 (54%)	0.133	0.7160
Height (cm)	115.40 ± 9.38	111.90 ± 6.61	2.065	0.0420*
Weight (kg)	21.34 ± 4.15	20.78 ± 4.60	0.592	0.5557
BMI (kg/m^2^)	15.93 ± 1.80	16.50 ± 2.47	1.201	0.2330
OAHI (events/h)	0.18 ± 0.13	8.94 ± 4.56	11.840	<0.0001****
LaSO_2_ (%)	90.58 ± 3.42	70.68 ± 14.63	8.206	<0.0001****
SBP (mmHg)	101.60 ± 6.96	102.40 ± 10.76	0.373	0.7098
DBP (mmHg)	61.26 ± 7.57	63.78 ± 7.77	1.522	0.1316
Hb (g/dl)	12.72 ± 0.77	12.39 ± 0.99	1.661	0.1007
ALT (U/L)	11.87 ± 3.99	12.91 ± 6.20	0.904	0.3684
AST (U/L)	28.66 ± 5.58	30.60 ± 6.66	1.451	0.1504
CK-MB (U/L)	24.36 ± 8.59	25.43 ± 4.48	0.757	0.4514
UA (μmol/L)	277.40 ± 62.25	260.10 ± 55.99	1.373	0.1732
Scr (μmol/L)	45.82 ± 9.96	51.85 ± 7.32	3.276	0.0015**
BUN (mmol/L)	5.12 ± 1.35	4.96 ± 1.34	0.572	0.5690
TG (mmol/L)	1.01 ± 0.43	1.21 ± 0.59	1.690	0.0947
TC (mmol/L)	4.29 ± 0.59	4.60 ± 0.66	2.306	0.0235*
Glucose (mmol/L)	5.13 ± 0.91	5.14 ± 0.73	0.099	0.9218

HC, healthy control; BMI, body mass index; OAHI, obstructive apnea hypopnea index; LaSO_2_, lowest oxygen saturation; SBP, systolic blood pressure; DBP, diastolic blood pressure; Hb, hemoglobin; ALT, alanine aminotransferase; AST, aspartate aminotransferase; CK-MB, creatine kinase-MB; UA, uric acid; BUN, blood urea nitrogen; TG, triglyceride; TC, total cholesterol.

* *p < *0.05, ** *p* < 0.01, **** *p* < 0.0001.

### 3.2. High-throughput sequencing results

**Figure 2:**
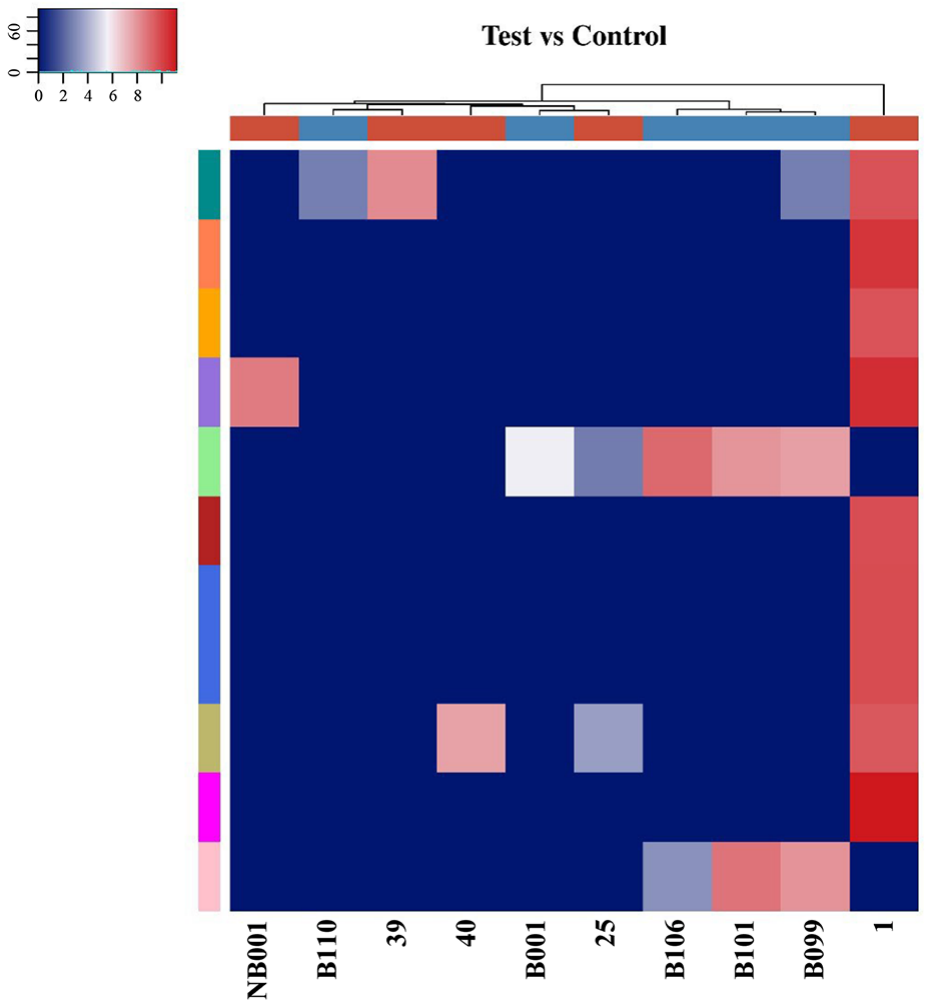
The difference of tsRNAs expression between OSAHS children and healthy children was analyzed by hierarchical clustering heat maps. Each row represented a tsRNA and each column represented a sample. The 25, 1, 39, NB001, and 40 were plasma specimens of children with OSAHS. The B009, B110, B106, B001, and B101 were plasma specimens of normal children. The degree of difference gradually increases from blue to red.

**Figure 3:**
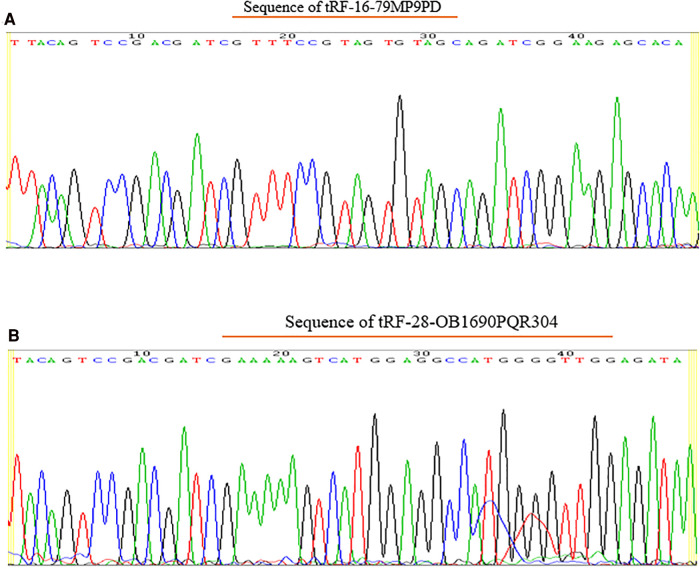
Sequencing results of qRT-PCR products of tRF-16-79MP9PD (**A**) and tRF-28-OB1690PQR304 (**B**).

Among the 10 sequencing libraries, the average number of original reads was about 6.69 million and the average reads ratio of high-quality bases was about 96.97%. Among them, microRNAs accounted for the highest proportion, with an average read number of 935,592.4 (17.64%). The average reads of mature tRNA and precursor tRNA were 156,419.3 (3.04%) and 5,973.1 (0.12%). A total of 499 tsRNAs were detected, of which 66 were recorded in the tRF database (Figure 1). Through the comparison between the two groups, there were 11 tsRNAs that were significantly differentially expressed, of which two tsRNAs were up-regulated and the remaining 9 tsRNAs were down-regulated (Figure 2). Taking into account the fold change, *P* value and tsRNAs expression difference between two groups, we selected tRF-16-79MP9PD and tRF-28-OB1690PQR304 as our research objects. After that, we designed primers for the two tsRNAs, and the sequencing results of the qRT-PCR products proved the specificity of the primers (Figure 3).

### 3.3. Expression levels of tRF-16-79mp9pd and tRF-28-OB1690PQR304

**Figure 4:**
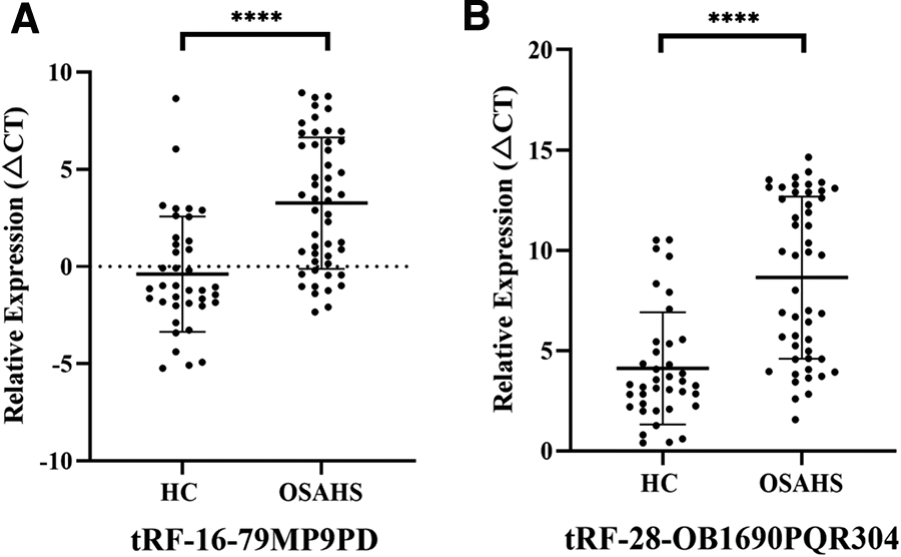
Expression of tRF-16-79MP9PD (**A**) (*t* = 5.289, 95% CI: 2.278–5.022) and tRF-28-OB1690PQR304 (**B**) (*t* = 5.886, 95% CI: 2.994–6.049) in patients with OSAHS. ****p < 0.0001; CI, Confidence Interval.

To further investigate the differential expression of tRF-16-79MP9PD and tRF-28-OB1690PQR304 in OSAHS children, we verified the expression of both tRFs in an extended cohort. As expected, our results showed that the expression levels of tRF-16-79MP9PD and tRF-28-OB1690PQR304 were significantly lower in patients with OSAHS than in controls (both, *P* < 0.0001), which was consistent with the results of high-throughput sequencing (Figure 4).

### 3.4. Potential diagnostic values of tRF-16-79mp9pd and tRF-28-OB1690PQR304

**Figure 5:**
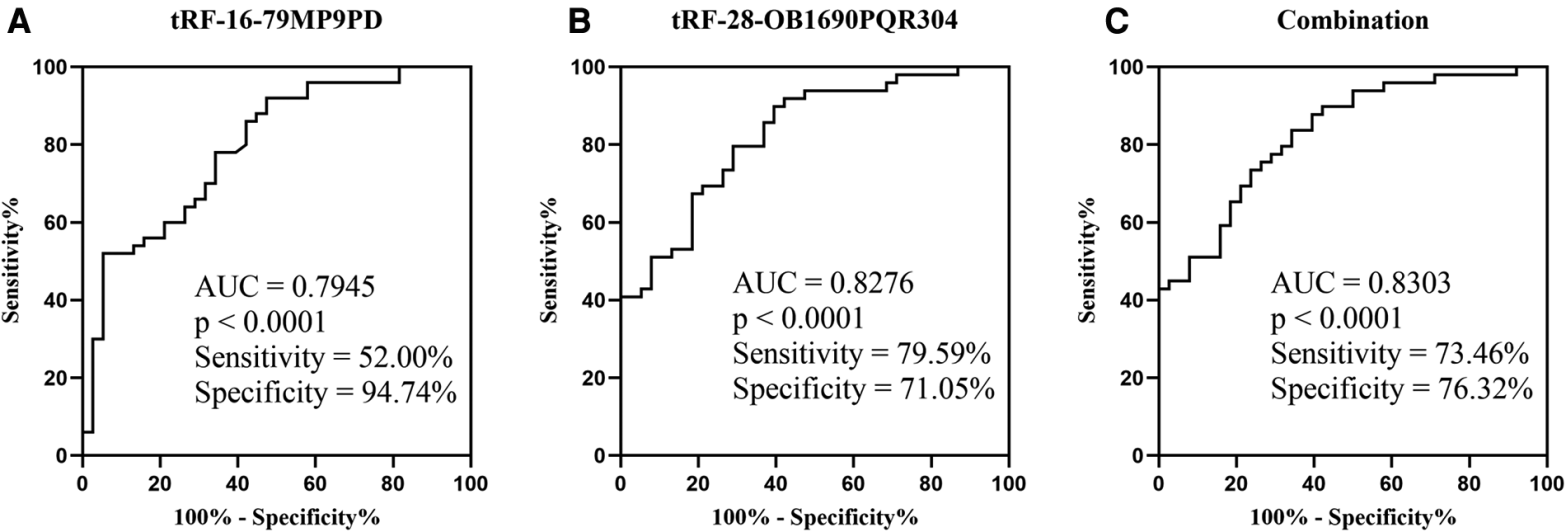
ROC of plasma tRF-16-79MP9PD (**A**) (SE: 0.0478, 95% CI: 0.7007–0.8882), tRF-28-OB1690PQR304 (**B**) (SE: 0.0433, 95% CI: 0.7428–0.9124) and the combination (**C**) (SE: 0.0428, 95% CI: 0.7465–0.9141; OR: tRF-16-79MP9PD: 1.1219, tRF-28-OB1690PQR304: 0.6419). SE, Standard Error; OR, Odds Ratio.

The ROC analysis revealed that the AUCs of tRF-16-79MP9PD and tRF-28-OB1690PQR304 were 0.7945 with 52% sensitivity and 94.74% specificity (Figure 5A), 0.8276 with 79.59% sensitivity and 71.05% specificity, respectively (Figure 5B). Furthermore, the AUC of the combination reached 0.8303 with 73.46% sensitivity and 76.32% specificity (Figure 5C).

### 3.5. Correlations between tRFs and clinical parameters

**Figure 6:**
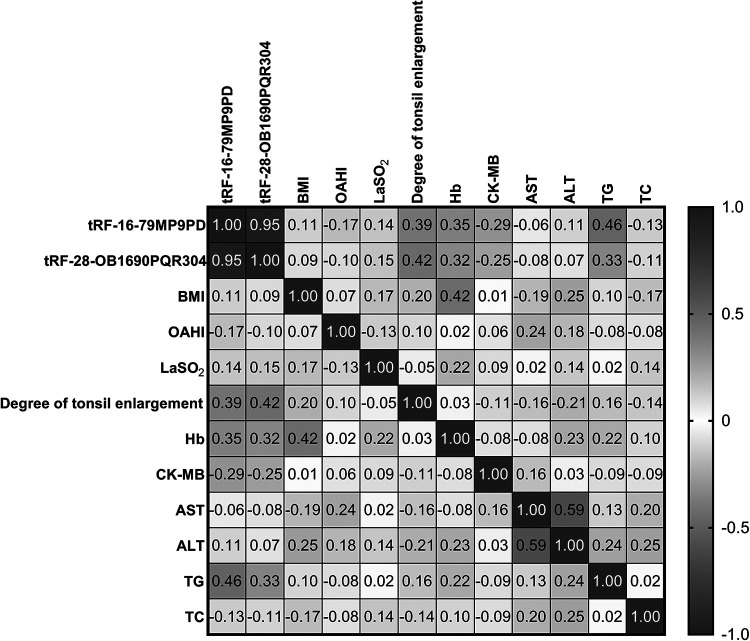
The visual correlation heat map of correlation between tRF-16-79MP9PD and tRF-28-OB1690PQR304 and the clinical parameters.

**Figure 7:**
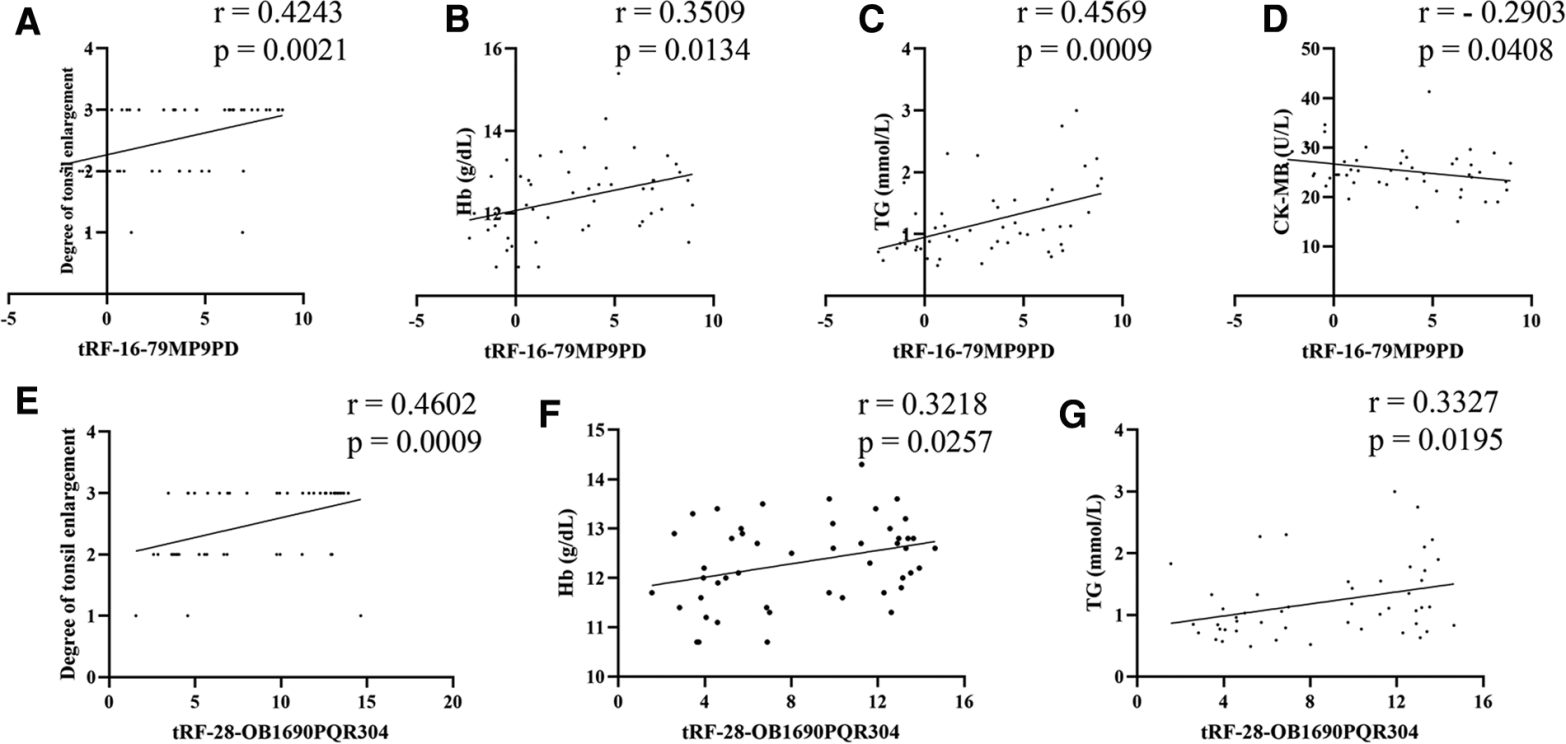
Correlation between tRF-16-79MP9PD and the degree of tonsil enlargement (**A**), Hb (**B**), TG (**C**) and CK-MB (**D**) level, tRF-28-OB1690PQR304 and the degree of tonsil enlargement (**E**), Hb (**F**), TG (**G**). Hb, hemoglobin, TG, triglyceride, CK-MB, creatine kinase-MB.

Firstly, we used simple linear correlation to analyze the correlation between tRF-16-79MP9PD and tRF-28-OB1690PQR304 and the clinical parameters we collected. The visual correlation heat map (Figure 6) showed the correlation between all parameters. We found that the level of tRF-16-79MP9PD was significantly correlated with the degree of tonsil enlargement, Hb, TG and CK-MB level (Figures 7A–D) while tRF-16-79MP9PD was correlated with the degree of tonsil enlargement, Hb and TG (Figures 7E–G).

Subsequently, we performed multiple linear regression using the regression method. The results showed that degree of tonsil enlargement, Hb and TG levels were related to the tRF-16-79MP9PD expression (Table 3) whose mathematic model was *y* = −10.309 + 0.902*x*_1_ + 1.887*x*_2_ − 0.160*x*_3_ + 1.656*x*_4_. Differently, Hb and the degree of tonsil enlargement were related to tRF-28-OB1690PQR304 (Table 4) whose mathematic model was *y* = −14.383 + 1.353*x*_1_ + 2.503*x*_2_.

**Table 3:** Multivariate linear regression analysis of plasma tRF-16-79MP9PD level and clinical parameters.

Associated parameters	B	SE	95% CI	*t*	*P-*value
Hb (*x*_1_)	0.902	0.421	(0.053, 1.752)	2.141	0.038
TG (*x*_2_)	1.887	0.684	(0.509, 3.266)	2.759	0.008
CK-MB (*x*_3_)	−0.160	0.087	(−0.336, 0.016)	−1.828	0.074
Degree of tonsil enlargement (*x*_4_)	1.656	0.643	(0.360, 2.953)	2.575	0.013
Constant	−10.309	5.999	(−22.400, 1.782)	−1.718	0.093

**Table 4:** Multivariate linear regression analysis of plasma tRF-28-OB1690PQR304 level and clinical parameters.

Associated parameters	B	SE	95% CI	*t*	*P-*value
Hb (*x*_1_)	1.353	0.615	0.114, 2.592	2.199	0.033
Degree of tonsil enlargement (*x*_2_)	2.503	0.845	0.802, 4.204	2.963	0.005
Constant	−14.383	7.690	−29.871, 1.104	−1.870	0.068

## 4. Discussion

As one of the most serious diseases among childhood sleep disordered breathing, OSAHS is characterized by hypoventilation, decreased blood oxygen saturation and disrupted sleep structure (24). Currently, guidelines recommend PSG as a standard diagnostic method for pediatric OSAHS. However, standard PSG monitoring is limited by the equipment, operation, personnel and cost of standard PSG monitoring. Although many efforts have been made to explore biomarkers of OSAHS, there is currently no definite biomarker available in the clinic.

tsRNAs exist widely in tissues and cells of various organisms with tissue specificity and disease correlation and can play roles in cell proliferation, regulation of gene expression, RNA processing, modulation of DNA damage response and tumor suppression (25). tRFs can be expressed and measured in human body fluids such as serum, plasma and urine. In addition, they are not easily to be degraded by RNase due to their short length. Thus, tRFs have great potential to serve as a non-invasive biomarker for various diseases (18). Accumulating evidence has demonstrated tRFs serve as ideal biomarkers and therapeutic targets in a variety of diseases, such as prostate cancer (26), pancreatic ductal adenocarcinoma (27) and systemic lupus erythematosus (28).

To our knowledge, this study was the first to investigate the plasma tRFs levels in children with OSAHS. Specifically, we first analyzed general baseline data of the OSAHS and HC groups. The results showed that the height of children with OSAHS was lower than that of HC group, which was consistent with the research of Johnson C (29) and may due to the decrease of growth hormone secretion caused by poor sleep quality of children with OSAHS. Similarly, the two groups had statistical differences in Scr and TC, which was consistent with the studies of other scholars (30, 31). Secondly, we performed small RNA sequencing to detect the expression profile of tRFs in 5 pairs of OSAHS children and control specimens. The results showed that the expression of 11 tsRNAs was significantly different between the two groups. Two dysregulated tRFs: tRF-16-79MP9PD and tRF-28-OB1690PQR304 were screened out based on fold changes, *P* value and tsRNAs expression differences. Subsequently, qRT-PCR was performed in the extended cohort to verify the authenticity of the sequencing results. It was proved that the expression levels of tRF-16-79MP9PD and tRF-28-OB1690PQR304 were down-regulated in the OSAHS group compared with the HC group, which was consistent with the results of small RNA sequencing, indicating that the decreased expression of tRF-16-79MP9PD and tRF-28-OB1690PQR304 were closely related to OSAHS.

In addition, we further evaluated the diagnostic efficacy of tRF-16-79MP9PD and tRF-28-OB1690PQR304 by ROC curve analysis, and the results demonstrated that tRF-16-79MP9PD and tRF-28-OB1690PQR304 had high sensitivity and specificity to distinguish OSAHS from healthy children, processing AUCs of 0.7945 with 52% sensitivity and 94.74% specificity, 0.8276 with 79.59% sensitivity and 71.05% specificity, respectively. More importantly, the AUC of their combination increased to 0.8303 with 73.46% sensitivity and 76.32% specificity, suggesting the great potential of tRF-16-79MP9PD and tRF-28-OB1690PQR304 as diagnosis biomarkers for childhood OSAHS. At present, studies on OSAHS in children have not obtained clear biomarkers, and existing studies also have some limitations, such as lack of specificity and sensitivity analysis (32). In our study, the AUC of the joint diagnosis of two tRFs exceeds 0.83, and the sensitivity and specificity exceed 70%, which has certain clinical screening diagnostic value. As a clinical OSAHS screening indicator, tRF can serve as a new research direction, but its diagnostic value needs to be evaluated in multi-center studies with larger sample sizes.

Furthermore, we analyzed the correlation between tRFs levels and laboratory parameters in OSAHS children. We found that the expression levels of tRF-16-79MP9PD and tRF-28-OB1690PQR304 were closely related to the degree of tonsil enlargement. At the same time, we found that the expression level of tRF-16-79MP9PD was also significantly correlated with Hb, TG and CK-MB level. Multivariable linear regression analysis showed that Hb, TG and degree of tonsil enlargement were related to tRF-16-79MP9PD. In addition, tRF-28-OB1690PQR304 was significantly correlated with Hb and TG. The degree of tonsil enlargement and Hb were related to the tRF-28-OB1690PQR304 level through multivariable linear regression analysis. Thus, the plasma levels of tRF-16-79MP9PD and tRF-28-OB1690PQR304 are related to a variety of laboratory indicators in OSHAS. More experiments are needed to investigate the role of tRF-16-79MP9PD and tRF-28-OB1690PQR304 in OSAHS.

Nevertheless, there are still some limitations to our study. Firstly, the sample size of this study was 50 OSAHS children and 38 healthy controls. Further expansion of sample size is needed to evaluate the diagnostic value of tRFs. Secondly, this study did not explore the function of tRFs by combining the pathological and physiological mechanisms of OSAHS. In the future, we will explore the role of tRFs in the pathogenesis of OSAHS based on this research. Thirdly, this study was limited to children in Ningbo City. Considering regional and racial differences, future multicenter and larger sample size studies are needed to evaluate the value of tRF as a biomarker.

## 5. Conclusions

In conclusion, our study identified that the plasma levels of tRF-16-79MP9PD and tRF-28-OB1690PQR304 were down-regulated in 4- to 7-year-old children with OSAHS and were closely related to the clinical parameters of OSAHS children. tRF-16-79MP9PD and tRF-28-OB1690PQR304 may be used as a new potential biomarker for the diagnosis of pediatric OSAHS.

## Data Availability

The data presented in the study are deposited in the NCBI BioProject repository, accession number PRJNA862251.
